# Supplementary material for: Global relationships between body size and urban affinity across more than 30,000 plant and animal species
Source: eLife. 2026 Aug 11;14:RP109047. doi: 10.7554/eLife.109047 (PMC13461149; doi:10.7554/eLife.109047)
Supplement: Peer review history

# Editor report: eLife Assessment

**DOI:** 10.7554/eLife.109047.3.sa0

**Authors:** Bernhard Schmid

**Affiliations:** University of Zurich Switzerland

**Keywords:** Important, Solid

This study provides an **important** assessment of how body size influences the occurrence of macro-organisms in urban areas across the globe. Size in most plants, but only some animal families, was positively associated with urban affinity. The data set is impressive and the strength of evidence **solid**.

# Referee report: Reviewer #2 (Public review)

**DOI:** 10.7554/eLife.109047.3.sa1

I have completed a thorough review of this paper, which seeks to use the large datasets of species occurrences available through GBIF to estimate variation in how large numbers of plant and animal species are associated with urbanization throughout the world, describing what they call the "species urbanness distribution" or SUD. They explore how these SUDs differ between regions and different taxonomic levels. They then calculate a measure of urban tolerance and seek to explore whether organism size predicts variation in tolerance among species and across regions.

The study is impressive in many respects. Over the course of several papers, Callaghan and coauthors have been leaders in using "big [biodiversity] data" to create metrics of how species' occurrence data are associated with urban environments, and in describing variation in urban tolerance among taxa and regions. This work has been creative, novel, and it has pushed the boundaries of understanding how urbanization affects a wide diversity of taxa. The current paper takes this to a new level by performing analyses on over 94000 observations from >30,000 species of plants and animals, across more than 370 plant and animal taxonomic families. All of these analyses were focused on answering two main questions:

(1) What is the shape of species' urban tolerance distributions within regional communities?

(2) Does body size consistently correlate with species' urban tolerance across taxonomic groups and biogeographic contexts?

Overall, I think the questions are interesting and important, the size and scope of the data and analyses are impressive, and this paper has a potentially large contribution to make in pushing forward urban macroecology specifically and urban ecology and evolution more generally.

Despite my enthusiasm for this paper and its potential impact, there are aspects that could be improved, and I believe the paper requires major revision.

Some of these revisions ideally involve being clearer about the methodology or arguments being made. In other cases, I think their metrics of urban tolerance are flawed and need to be rethought and recalculated, and some of the conclusions are inaccurate. I hope the authors will address these comments carefully and thoroughly. I recognize that there is no obligation for authors to make revisions. However, revising the paper along the lines of the comments made below would increase the impact of the paper and its clarity to a broad readership.

Major Comments:

(1) Subrealms

Where does the concept of "subrealms" come from? No citation is given, and it could be said that this sounds like an idea straight out of Middle Earth. How do subrealms relate to known bioclimatic designations like Koppen Climate classifications, which would arguably be more appropriate? Or are subrealms more socio-ecologically oriented? From what I can tell, each subrealm lumps together climatically diverse areas. It might be better and more tractable to break things in terms of continents, as the rationale for subrealms is unclear, and it makes the analyses and results more confusing. The authors rationalized the use of subrealms to account for potential intraspecific differences in species' response to urbanization, but that is never a core part of the questions or interpretation in the paper, and averaging across subrealms also accounts for intraspecific variation. Another issue with using the subrealm approach is that the authors only included a species if it had 100 observations in a given subrealm, leading to a focus on only the most common species, which may be biased in their SUD distribution. How many more species would be included if they did their analysis at the continental or global scale, and would this change the shape of SUDs?

(2) Methods - urban score

The authors describe their "urban score" as being calculated as "the mean of the distribution of VIIRS values as a relative species-specific measure of a response to urban land cover."

I don't understand how this is a "relative species-specific measure". What is it relative to? Figures S4 and S5 show the mean distribution of VIIRS for various taxa, and this mean looks to be an absolute measure. Mean VIIRS for a given species would be fine and appropriate as an "urban score", but the authors then state in the next sentence: "this urban score represents the relative ranking of that species to other species in response to urban land cover".

That doesn't follow from the description of how this is calculated. Something is missing here. Please clarify and add an explicit equation for how the urban score is calculated because the text is unclear and confusing.

(3) Methods - urban tolerance

How the authors are defining and calculating tolerance is unclear, confusing, and flawed in my opinion.

Tolerance is a common concept in ecology, evolution, and physiology, typically defined as the ability for an organism to maintain some measure of performance (e.g., fitness, growth, physiological homeostasis) in the presence versus absence of some stressor. As one example, in the herbivory literature, tolerance is often measured as the absolute or relative difference in fitness of plants that are damaged versus undamaged (e.g., https://doi.org/10.1111/j.1558-5646.2008.00458.x).

On line 309, after describing the calculation of urban scores across subrealms, they write: "Therefore, a species could be represented across multiple subrealms with differing measures of urban tolerance (Fig. S4). Importantly, this continuous metric of urban tolerance is a relative measure of a species' preference, or affinity, to urban areas: it should be interpreted only within each subrealm".

This is problematic on several fronts. First, the authors never define what they mean by the term "tolerance". Second, they refer to urban tolerance throughout the paper, but don't describe the calculation until lines 315-319, where they write (text in [] is from the reviewer):

"Within each subrealm, we further accounted for the potential of different levels of urbanization by scaling each species' urban score by subtracting the mean VIIRS of all observations in the subrealm (this value is hereafter referred to as urban tolerance). This 'urban tolerance' (Fig. S5) value can be negative - when species under-occupy urban areas [relative to the average across all species] suggesting they actively avoid them-or positive-when species over-occupy urban areas [relative to the average across all species] suggesting they prefer them (i.e., ranging from urban avoiders to urban exploiters, respectively).

They are taking a relativized urban score and then subtracting the mean VIIRS of all observations across species in a subrealm. How exactly one interprets the magnitude isn't clear and they admit this metric is "not interpretative across subrealms".

This is not a true measure of tolerance, at least not in the conventional sense of how tolerance is typically defined. The problem is that a species distribution isn't being compared to some metric of urbanness, but instead it is relative to other species' urban scores, where species may, on average, be highly urban or highly nonurban in their distribution, and this may vary from subrealm to subrealm. A measure of urban tolerance should be independent of how other species are responding, and should be interpretable across subrealms, continents, and the globe.

I propose the authors use one of two metrics of urban tolerance:

(i) Absolute Urban Tolerance = Mean VIIRS of species_i - Mean VIIRS of city centers

Here, the mean VIIRS of city centers could be taken from the center of multiple cities throughout a subrealm, across a continent, or across the world. Here, the units are in the original VIIRS units where 0 would correspond to species being centered on the most extreme urban habitats, and the most extreme negative values would correspond to species that occupy the most non-urban habitats (i.e., no artificial light at night). In essence, this measure of tolerance would quantify how far a species' distribution is shifted relative to the most highly urbanized habitat available.

(ii) % Urban Tolerance = (Mean VIIRS of species_i - Mean VIIRS of city centers)/MeanVIIRS of city centers * 100%

This metric provides a % change in species mean VIIRS distribution relative to the most urban habitats. This value could theoretically be negative or positive, but will typically be negative, with -100% being completely non-urban, and 0% being completely urban tolerant.

Both of these metrics can be compared across the world, as it would provide either absolute (equation 1) or relative (equation 2) metrics of urban tolerance that are comparable and easily interpretable in any region.

In summary, the definition of tolerance should be clear, the metric should be a true measure of tolerance that is comparable across regions, and an equation should be given.

(4) Figure 1: The figure does not stand alone. For example, what is the hypothesis for thermophily or the temperature-size rule? The authors should expand the legend slightly to make the hypotheses being illustrated clearer.

(5) SUDs: I don't agree with the conclusion given on line 83 ("pattern was consistent across subrealms and several taxonomic levels") or in the legend of Figure 2 ("there were consistent patterns for kingdoms, classes, and orders, as shown by generally similar density histograms shapes for each of these").

The shapes of the curves are quite different, especially for the two Kingdoms and the different classes. I agree they are relatively consistent for the different taxonomic Orders of insects.

Comments on revised version:

I believe their response is thorough and thoughtful. I still disagree with them on some fundamental points of their methodology. However, I would prefer to let my review and their response stand as is. This will allow engaged readers to see both sides of the arguments and judge for themselves whether they believe the revisions are sufficient and if my concerns are valid.

# Author comment: Author response

**DOI:** 10.7554/eLife.109047.3.sa2

**Authors:** Corey T Callaghan, Diana E Bowler, Vaughn Shirey, Brittany M Mason, Laura H Antao, Ingmar Staude, John H Wilshire, Thomas Merckx

**Affiliations:** Department of Wildlife Ecology and Conservation, Fort Lauderdale Research and Education Center, Institute of Food and Agricultural Sciences, University of Florida Florida United States; UK Centre for Ecology and Hydrology Wallingford United Kingdom; Department of Biology, Georgetown University Washington, DC United States; Marine and Environmental Biology Section, Department of Biological Sciences, University of Southern California Los Angeles United States; Department of Wildlife Ecology and Conservation, Fort Lauderdale Research and Education Center, Institute of Food and Agricultural Sciences, University of Florida Florida United States; Research Centre for Ecological Change, Faculty of Biological and Environmental Sciences, University of Helsinki Helsinki Finland; Department of Biology, University of Turku Finland; Institute of Biology, Leipzig University Leipzig Germany; German Centre of Integrative Biodiversity Research (iDiv) Halle-Jena-Leipzig Germany; Department of Ecology and Evolutionary Biology, Yale University Connecticut United States; Center for Biodiversity and Global Change, Yale University Connecticut United States; Wildness, Biodiversity and Ecosystems under Change (WILD), Department of Biology, Vrije Universiteit Brussel Brussels Belgium

The following is the authors’ response to the original reviews.

**eLife Assessment**

This study provides an important assessment of how body size influences the occurrence of macro-organisms in urban areas across the globe. Size in most plants, but only some animal families, was positively associated with urban tolerance. The data set is impressive, but the evidence for broad-scale conclusions is incomplete due to methodological issues that need to be resolved.

We have substantially revised the manuscript to resolve the methodological issues raised, including clarifying the definition, calculation, and interpretation of urban affinity (formerly named urban tolerance), and tightening the scope of our conclusions to align directly with the evidence presented.

**Public Reviews:**

**Reviewer #1 (Public review):**

Summary:

The authors integrate multiple large databases to test whether body sizes were positively associated with which species tolerate urban areas. In general, many plant families showed a positive association between body size and urban tolerance, whereas a smaller, though still non-trivial, percentage of animal families showed the same pattern. Notably, the authors are careful in the interpretation of their findings and provide helpful context for the ways that this analysis can be generative in shaping new hypotheses and theory around how urbanization influences biodiversity at large. They are careful to discuss how body size is an important trait, but the absence of a relationship between body size and urban tolerance in many families suggests a variety of other traits undergird urban success.

We appreciate this thoughtful and balanced assessment of our work and fully agree with the reviewer’s interpretation. In particular, we share the view that the heterogeneous and often weak association between body size and urban affinity across many families is an important result in its own right, underscoring that no single trait is likely to explain urban success across the tree of life. As the reviewer notes, our intention was not to present body size as a universal predictor, but rather as a widely available, integrative trait that can help reveal where general patterns do and do not emerge. We view the lack of a consistent relationship in many families as strong motivation for future work that explicitly integrates additional functional traits and ecological contexts, and we have clarified this perspective in the revised manuscript.

Strengths:

The authors aggregated a large dataset, but they also applied robust filters to ensure they had an adequate and representative number of detections for a given species, family, geography, etc. The authors also applied their analysis at multiple taxonomic scales (family and order), which allowed for a better interpretation of the patterns in the data and at what taxonomic scale body size might be important.

We thank the reviewer for highlighting these strengths of the study. Considerable effort went into assembling, harmonizing, and filtering these data across taxa, regions, and taxonomic resolutions, and we were deliberate in applying conservative thresholds to ensure that species-level urban affinity estimates were based on adequate and comparable sampling. We hope that, beyond the specific results presented here, the compiled dataset and analytical framework will serve as a valuable resource for future studies aiming to explore additional traits, taxa, or mechanisms underlying species’ responses to urbanization.

Weaknesses:

My main concern is that it is not fully clear how the measure of body size might influence the result. The authors were unable to obtain consistent measures of body size (mean, median, maximum, or sex variation). This, of course, could be very consequential as means and medians can differ quite a bit, and they certainly will differ substantially from a maximum. And of course, sex differences can be marked in multiple directions or absent altogether. The authors do note that they selected the measure that was most common in a family, but it was not clear whether species in that family that did not have that measure were removed or not. This could potentially shape the variability in the dataset and obscure true patterns. This may require additional clarity from the authors and is also a real constraint in compiling large data from disparate sources.

We appreciate this important point and agree that heterogeneity in how body size is measured (e.g., mean vs. maximum values, sex-specific measures) is a real but unavoidable challenge when compiling organismal trait data across such a broad taxonomic scope. We would like to clarify that our analytical approach was explicitly designed to minimize the influence of this heterogeneity rather than ignore it. Specifically, for each family we retained all species for which at least one body size estimate was available, rather than removing species that lacked a particular measurement type. When multiple body size measures existed for a species, we selected the measurement type that was most commonly available within that family in order to maximize comparability among species while retaining sample size. Importantly, differences among body size measurement types (including units, measurement detail, and whether values reflected means, maxima, or sex-specific estimates) were further accounted for by (i) log-transforming all body size values and (ii) centering and scaling body size values within each measurement type, which was included as a random effect in the hierarchical models. This approach reduces the influence of systematic differences among measurement types on estimated relationships with urban affinity. We have added a sentence to the methods clarifying that species with a single measurement type were not removed from analyses:

“Importantly, this procedure did not result in the exclusion of species lacking a particular body size measurement type; rather, all species with at least one available body size estimate were retained, with measurement heterogeneity explicitly accounted for through hierarchical modeling.”

We agree that variation in body size definitions may still contribute residual noise and potentially obscure weak relationships, and we now emphasize this more clearly as a limitation of large-scale trait syntheses. However, because our primary inference focuses on the presence, absence, and direction of size–urban affinity relationships across families, rather than precise effect sizes, we believe our approach provides a robust and conservative test of whether body size consistently predicts urban affinity across taxa. We highlight this point in the limitations section of our manuscript:

“One important limitation of our synthesis is the heterogeneity in how body size is measured across taxa, including differences among mean, maximum, and sex-specific estimates. While our analytical framework explicitly accounts for this variation through transformation, scaling, and hierarchical modeling with random intercepts (see Methods), residual measurement noise may still obscure weak size–urban affinity relationships. This challenge is inherent to large-scale trait syntheses that integrate data from disparate sources, and highlights the need for continued efforts to standardize trait databases and expand the availability of harmonized organismal trait data across the tree of life.”

**Reviewer #2 (Public review):**

I have completed a thorough review of this paper, which seeks to use the large datasets of species occurrences available through GBIF to estimate variation in how large numbers of plant and animal species are associated with urbanization throughout the world, describing what they call the "species urbanness distribution" or SUD. They explore how these SUDs differ between regions and different taxonomic levels. They then calculate a measure of urban tolerance and seek to explore whether organism size predicts variation in tolerance among species and across regions.

The study is impressive in many respects. Over the course of several papers, Callaghan and coauthors have been leaders in using "big [biodiversity] data" to create metrics of how species' occurrence data are associated with urban environments, and in describing variation in urban tolerance among taxa and regions. This work has been creative, novel, and it has pushed the boundaries of understanding how urbanization affects a wide diversity of taxa. The current paper takes this to a new level by performing analyses on over 94000 observations from >30,000 species of plants and animals, across more than 370 plant and animal taxonomic families. All of these analyses were focused on answering two main questions:

(1) What is the shape of species' urban tolerance distributions within regional communities?

(2) Does body size consistently correlate with species' urban tolerance across taxonomic groups and biogeographic contexts?

We thank the reviewer for their careful reading of the manuscript and for this generous and accurate summary of the study’s aims, scope, and contributions. We appreciate the recognition of our group’s broader body of work using large biodiversity databases to quantify species’ associations with urban environments, and we are grateful for the reviewer’s acknowledgement that this study extends those efforts to an unprecedented taxonomic and geographic scale. We agree with the reviewer’s articulation of the two core questions motivating the paper, and we have revised the manuscript to ensure that these questions are stated clearly and addressed consistently throughout.

Overall, I think the questions are interesting and important, the size and scope of the data and analyses are impressive, and this paper has a potentially large contribution to make in pushing forward urban macroecology specifically and urban ecology and evolution more generally.

Thanks! We see this work as an effort to move beyond species-by-species descriptions of urban responses toward a community- and distribution-level perspective, where the shape of species’ urban associations themselves becomes an object of study. By framing species’ distributions along an urbanization gradient as a collective property of regional species pools, our approach opens a complementary way of thinking about how urbanization filters biodiversity.

Despite my enthusiasm for this paper and its potential impact, there are aspects that could be improved, and I believe the paper requires major revision.

Some of these revisions ideally involve being clearer about the methodology or arguments being made. In other cases, I think their metrics of urban tolerance are flawed and need to be rethought and recalculated, and some of the conclusions are inaccurate. I hope the authors will address these comments carefully and thoroughly. I recognize that there is no obligation for authors to make revisions. However, revising the paper along the lines of the comments made below would increase the impact of the paper and its clarity to a broad readership.

We appreciate the detailed comments provided and have addressed each point in turn - see detailed responses below. We took these concerns seriously and undertook a substantial revision of the manuscript. In summary, we clarified the conceptual framing of “urban tolerance” (now referred to as “urban affinity”), explicitly defined the metric and its interpretation, added equations and a step-by-step methodological roadmap, and expanded justification for our regional stratification. Where appropriate, we refined language in the Results and Discussion to ensure conclusions are tightly aligned with what the metric can and cannot support. We agree that these revisions materially improve the clarity, rigor, and interpretability of the study, and we appreciate the reviewer’s perspective on how doing so strengthens the paper’s contribution and accessibility to a broad readership.

Major Comments:

(1) Subrealms

Where does the concept of "subrealms" come from? No citation is given, and it could be said that this sounds like an idea straight out of Middle Earth. How do subrealms relate to known bioclimatic designations like Koppen Climate classifications, which would arguably be more appropriate? Or are subrealms more socio-ecologically oriented? From what I can tell, each subrealm lumps together climatically diverse areas. It might be better and more tractable to break things in terms of continents, as the rationale for subrealms is unclear, and it makes the analyses and results more confusing. The authors rationalized the use of subrealms to account for potential intraspecific differences in species' response to urbanization, but that is never a core part of the questions or interpretation in the paper, and averaging across subrealms also accounts for intraspecific variation. Another issue with using the subrealm approach is that the authors only included a species if it had 100 observations in a given subrealm, leading to a focus on only the most common species, which may be biased in their SUD distribution. How many more species would be included if they did their analysis at the continental or global scale, and would this change the shape of SUDs?

We thank the reviewer for raising this point and agree that the rationale for using subrealms required clearer explanation. Next to allowing potential intraspecific differences in urban affinity across regions, our subrealm-based approach also provides a practical way to partition global biodiversity into ecologically meaningful regional assemblages while maintaining sufficient sample sizes for analysis. Urban affinity is likely to vary geographically within species due to differences in climate, habitat availability, urban form, and evolutionary history. By calculating urban affinity within subrealms rather than globally, our approach allows species to exhibit region-specific urban affinities while ensuring that comparisons are made among species co-occurring within the same regional ecological context. We have substantially revised the Methods to explicitly define subrealms, cite their origin, and clarify why this spatial stratification is appropriate for our study:

“Accounting for geographic context through subrealm stratification

To account for geographic heterogeneity in both species’ distributions and the baseline levels of urbanization, we stratified our analyses by global biogeographic subrealms (N=52; Fig. S1). Subrealms represent an intermediate hierarchical level within the One Earth [82] (https://www.oneearth.org/bioregions/) bioregionalization framework, grouping the 185 terrestrial bioregions into broader units that reflect shared species pools and ecological contexts while maintaining meaningful regional structure. This scale represents a practical compromise between analyzing data at the finer bioregion level (which would result in many regions with insufficient observations for robust analysis) and broader classifications such as continents or the 14 biogeographic realms, which aggregate ecologically distinct regions and species pools. This regionalization has been widely used in macroecological and biogeographic research to contextualize species–environment relationships because subrealms capture meaningful gradients in biotic assemblages that are not accounted for by climatic classifications alone [83,84].

This stratification allows species’ associations with urban environments to be interpreted relative to the environments available within the regions they occupy. This is important, as previous work has shown that species’ responses to urbanization are constrained by biogeographic context, because regional species pools reflect shared evolutionary, ecological, and historical filters [23]. Previous work has also shown that urban associations among species are context-dependent, and interpreting species’ responses without accounting for regional baselines conflates availability of urban environments with species’ affinity to them. This distinction is critical because identical levels of urbanization (e.g., VIIRS radiance) can have different ecological meanings across regions with different species pools and land-use histories. It avoids conflating species’ urban affinity with global differences in urban availability.”

We chose subrealms rather than Köppen climate classifications or continental units because our objective was not to partition species by climatic similarity per se, but to evaluate species’ associations with urban environments relative to the ecological and biogeographic contexts in which they occur. Climatic classifications such as Köppen are highly effective for addressing climate–species relationships, but they do not explicitly capture differences in species pools, evolutionary history, or land-use legacies that strongly shape how species interact with urbanization. Likewise, continents often aggregate ecologically disparate regions and species pools, potentially obscuring meaningful variation in baseline urbanization and species’ realized distributions.

Importantly, urban affinity in our framework is a relative, context-dependent metric, explicitly interpreted within regions. Identical levels of urbanization (e.g., VIIRS radiance values) can have different ecological meanings across regions with distinct species pools, land-use histories, and settlement patterns. Stratifying analyses by subrealm therefore avoids conflating species’ affinity to urban environments with global or continental differences in the availability and intensity of urban land cover. We have clarified this distinction and motivation in the revised Methods (see responses below).

Regarding the concern that requiring ≥100 observations per species per subrealm biases analyses toward common species: we agree that this threshold focuses the analysis on well-sampled species. This choice was intentional and follows previous work showing that such cutoffs are necessary to robustly characterize species’ responses to urbanization using occurrence data. While a global or continental analysis would indeed include additional, rarer species, it would also substantially increase uncertainty and conflate species’ responses across ecologically distinct contexts. Our study is therefore best interpreted as a macroecological synthesis of common species, which are also the taxa that disproportionately structure urban communities and drive the shape of Species Urbanness Distributions (SUDs). We now clarify this scope and limitation more explicitly in the introduction:

“Our aim is to identify broad, cross-taxonomic patterns in species’ urban affinity at a global scale, rather than to resolve the specific causal mechanisms driving urban success or failure within individual taxa or cities.”.

As well as in the discussion:

“Our synthesis complements taxon-specific, presence–absence trait studies by identifying broad, cross-taxonomic patterns that can motivate and contextualize more mechanistic analyses [17,23].”

Finally, while alternative spatial stratifications are possible, the central patterns we report particularly the skewed shape of SUDs—are robust to the use of regional context rather than absolute global metrics. Exploring how SUDs change under different spatial frameworks (e.g., continents, climate zones) is an interesting avenue for future work, but we feel is beyond the scope of the present study.

(2) Methods - urban score

The authors describe their "urban score" as being calculated as "the mean of the distribution of VIIRS values as a relative species specific measure of a response to urban land cover."

I don't understand how this is a "relative species-specific measure". What is it relative to? Figures S4 and S5 show the mean distribution of VIIRS for various taxa, and this mean looks to be an absolute measure. Mean VIIRS for a given species would be fine and appropriate as an "urban score", but the authors then state in the next sentence: "this urban score represents the relative ranking of that species to other species in response to urban land cover".

We agree that the wording in the original manuscript was unclear and conflated two distinct steps in the workflow. We have now revised the Methods to clearly distinguish between (i) the urban score, which is an absolute, descriptive summary of the mean VIIRS radiance associated with a species’ occurrence locations, and (ii) urban affinity, which is the relative, region-specific metric derived from the urban score. Specifically, we rewrote the methods to have distinct steps as subheadings, as follows: (1) urban score; (2) subrealms and why; (3) urban affinity. In the revised Methods, we explicitly define the urban score:

“an absolute descriptive summary of the urbanization levels associated with a species’ occurrence locations within a given subrealm”.

We no longer describe the urban score itself as “relative” or as a ranking among species. Relative comparisons among species arise only in the subsequent step, where species-specific urban scores are expressed relative to the regional background level of urbanization within each subrealm to derive urban affinity.

We refer the Reviewer to the revised version which we feel is much clearer (lines 428-479)!

That doesn't follow from the description of how this is calculated. Something is missing here. Please clarify and add an explicit equation for how the urban score is calculated because the text is unclear and confusing.

The previous response, where we discuss the description, hopefully clarifies this. Further, we have revised the Methods to clearly define the urban score and to include an explicit equation. In the revised manuscript, the urban score for species s is calculated as the mean VIIRS radiance across all occurrence locations of that species:

$$\displaystyle  U_{s, r}=\frac{1}{n_{s, r}} \sum_{i=1}^{n_{s, r}} L_{i}$$

where n_s_is the number of GBIF occurrence records for species s, and L_i_ is the VIIRS nighttime lights radiance value extracted at the location of occurrence i. We also clarify in the Methods that this urban score is an absolute summary statistic of observed urbanization at species occurrence locations

(3) Methods - urban tolerance

How the authors are defining and calculating tolerance is unclear, confusing, and flawed in my opinion.

Tolerance is a common concept in ecology, evolution, and physiology, typically defined as the ability for an organism to maintain some measure of performance (e.g., fitness, growth, physiological homeostasis) in the presence versus absence of some stressor. As one example, in the herbivory literature, tolerance is often measured as the absolute or relative difference in fitness of plants that are damaged versus undamaged (e.g., https://doi.org/10.1111/j.1558-5646.2008.00458.x).

On line 309, after describing the calculation of urban scores across subrealms, they write: "Therefore, a species could be represented across multiple subrealms with differing measures of urban tolerance (Fig. S4). Importantly, this continuous metric of urban tolerance is a relative measure of a species' preference, or affinity, to urban areas: it should be interpreted only within each subrealm". This is problematic on several fronts. First, the authors never define what they mean by the term "tolerance". Second, they refer to urban tolerance throughout the paper, but don't describe the calculation until, where they write (text in [] is from the reviewer): "Within each subrealm, we further accounted for the potential of different levels of urbanization by scaling each species' urban score by subtracting the mean VIIRS of all observations in the subrealm (this value is hereafter referred to as urban tolerance). This 'urban tolerance' (Fig. S5) value can be negative - when species under-occupy urban areas [relative to the average across all species] suggesting they actively avoid them-or positive-when species over-occupy urban areas [relative to the average across all species] suggesting they prefer them (i.e., ranging from urban avoiders to urban exploiters, respectively). They are taking a relativized urban score and then subtracting the mean VIIRS of all observations across species in a subrealm. How exactly one interprets the magnitude isn't clear and they admit this metric is "not interpretative across subrealms".

This is not a true measure of tolerance, at least not in the conventional sense of how tolerance is typically defined. The problem is that a species distribution isn't being compared to some metric of urbanness, but instead it is relative to other species' urban scores, where species may, on average, be highly urban or highly nonurban in their distribution, and this may vary from subrealm to subrealm. A measure of urban tolerance should be independent of how other species are responding, and should be interpretable across subrealms, continents, and the globe.

We thank the reviewer for this careful and important critique. We agree that the term “tolerance” is commonly used to describe the ability of an organism to maintain performance (e.g., fitness, growth, physiological homeostasis) in the presence of a stressor, and that our metric does not measure tolerance in this mechanistic or fitness-based sense. To address this directly and unambiguously, we have revised the manuscript to explicitly define the term “urban affinity” as opposed to urban tolerance.

In the revised Methods, we also reorganized and clarified the calculation of urban affinity, introduced explicit notation, and provided a formal equation. Specifically, we now define urban affinity for species s in subrealm r as:

$$\displaystyle A_{s, r}=U_{s, r}-\bar{U}_{r}$$

where U_s,r_is the mean VIIRS radiance across all occurrence locations of species s within subrealm r, and Ū_r_is the mean VIIRS radiance across all occurrence records of all species in that subrealm. This transformation centers species’ urban scores on the regional background level of urbanization, yielding a relative measure of spatial association with urban environments.

We agree with the reviewer that this metric is not interpretable as an absolute measure of affinity, and we now state this explicitly. Urban affinity values are, by construction, relative measures, interpretable only within subrealms, and they quantify whether a species tends to occur in more or less urbanized environments than is typical for that region. The magnitude of the metric therefore reflects deviation from the regional baseline, not a universal or global scale of urbanization, and is not intended to be compared directly across subrealms.

We respectfully disagree, however, that this makes the metric flawed. Rather, it reflects a deliberate analytical choice aligned with our research questions. Our goal was not to estimate absolute urban exposure or physiological performance, but to compare species’ realized spatial associations with urban environments within shared biogeographic contexts. Because baseline urbanization levels, settlement history, and species pools vary strongly across regions, a globally absolute metric would conflate species’ affinities with regional availability of urban environments. By contrast, a relative, region-centered metric allows meaningful comparisons among species that coexist within the same ecological and biogeographic setting. This approach follows a growing body of macroecological work that infers species’ environmental affinities from spatial distributions rather than direct performance measures (e.g., Callaghan et al. 2020; 2021; 2023), and we now cite these studies explicitly.

I propose the authors use one of two metrics of urban tolerance:

(i) Absolute Urban Tolerance = Mean VIIRS of species_i - Mean VIIRS of city centers Here, the mean VIIRS of city centers could be taken from the center of multiple cities throughout a subrealm, across a continent, or across the world. Here, the units are in the original VIIRS units where 0 would correspond to species being centered on the most extreme urban habitats, and the most extreme negative values would correspond to species that occupy the most non-urban habitats (i.e., no artificial light at night). In essence, this measure of tolerance would quantify how far a species' distribution is shifted relative to the most highly urbanized habitat available.

(ii) % Urban Tolerance = (Mean VIIRS of species_i - Mean VIIRS of city centers)/MeanVIIRS of city centers * 100%

This metric provides a % change in species mean VIIRS distribution relative to the most urban habitats. This value could theoretically be negative or positive, but will typically be negative, with -100% being completely non-urban, and 0% being completely urban tolerant.

Both of these metrics can be compared across the world, as it would provide either absolute (equation 1) or relative (equation 2) metrics of urban tolerance that are comparable and easily interpretable in any region.

In summary, the definition of tolerance should be clear, the metric should be a true measure of tolerance that is comparable across regions, and an equation should be given.

We thank the reviewer for this thoughtful and constructive suggestion, which raises an important conceptual issue regarding how “urban tolerance” should be defined and quantified. We agree that any such metric must be clearly defined, interpretable, and accompanied by an explicit equation, and we have revised the manuscript accordingly to clarify both our definition and its intended interpretation.

The alternative metrics proposed by the reviewer anchoring species’ distributions to city centers or to the most highly urbanized habitats represent a valid and intuitive absolute framing of urban tolerance. Indeed, a closely related approach was explored and evaluated in Callaghan et al. (2020; https://doi.org/10.1016/j.ecolind.2020.106905), where species’ occurrence-based urbanness scores derived from VIIRS night-time lights were compared against abundance-based estimates of urban tolerance using explicit urban–non-urban contrasts. That study further demonstrated that urbanness scores depend on the choice of spatial baseline (e.g., regional buffers around cities versus continental extents), and showed that different baselines capture complementary, but not identical, aspects of species–urban associations.

In the present study, we deliberately adopt a relative, regionally contextualized metric (now referred to as urban affinity), expressing each species’ mean VIIRS association relative to the background urbanization of the biogeographic subrealm in which it occurs. This choice reflects our goal of comparing species’ relative affinities to urban environments within shared ecological and biogeographic contexts. Importantly, identical VIIRS values can correspond to very different ecological conditions across regions, and anchoring all species to city centers or global urban maxima risks conflating species’ affinities with regional differences in urban availability and infrastructure.

We now make this distinction explicit throughout the manuscript, including by (i) defining urban affinity as a relative, occurrence-based measure of urban affinity (rather than physiological or fitness-based tolerance), (ii) providing an explicit equation for its calculation, and (iii) clarifying that these values are interpretable within, but not across, biogeographic subrealms. We view absolute, city-center–anchored metrics and relative, regionally normalized metrics as complementary approaches, each suited to different questions; the latter is most appropriate for the macroecological, comparative analyses pursued here.

(4) Figure 1: The figure does not stand alone. For example, what is the hypothesis for thermophily or the temperature-size rule? The authors should expand the legend slightly to make the hypotheses being illustrated clearer.

We now expanded the legend so that the figure and hypotheses presented can be understood based on just the figure and its legend; we did so by explaining the illustrated hypotheses as requested by the Reviewer. The figure legend now reads as follows:

“Fig. 1: Conceptual framework illustrating hypothesized mechanisms linking urban affinity to interspecific body-size shifts. These include dispersal and mobility constraints under habitat fragmentation [44,45], thermophily and the temperature–size rule driven by the urban heat island effect [15,30], size-biased competition and survival [94,95], and size-biased human preferences [64]. Urban fragmentation of habitat resources can select for increased mobility (e.g., larger butterflies) or reduced mobility (e.g., larger seeds) depending on isolation severity. Elevated urban temperatures favor thermophily, which often negatively correlates with size as it affects the heat balance via thermal inertia. Similarly, these higher temperatures generally favor smaller-bodied adult ectotherms because they accelerate development and reduce time available for growth (i.e., temperature-size rule). In plants, the increased CO_₂_ and nutrient availability associated with anthropogenic environments due to heating- and traffic-related CO2 emissions and eutrophication provides a competitive advantage to larger plant species, and human preferences too may favor larger species (e.g., tree-lined streets), whereas smaller species may be advantaged in colonizing built infrastructure.”

(5) SUDs: I don't agree with the conclusion given on line 83 ("pattern was consistent across subrealms and several taxonomic levels") or in the legend of Figure 2 ("there were consistent patterns for kingdoms, classes, and orders, as shown by generally similar density histograms shapes for each of these").

The shapes of the curves are quite different, especially for the two Kingdoms and the different classes. I agree they are relatively consistent for the different taxonomic Orders of insects.

We agree that our original wording overstated the similarity of distributions across taxa and regions. We have revised the text to clarify that the consistency we refer to pertains primarily to central tendencies rather than identical distributional shapes. To address this directly, we conducted additional analyses comparing urban affinity distributions across subrealms for taxonomic groups with the largest sample sizes. These results, now presented in new Supplementary Figures (Fig. S2-S4), show that while distributional shapes vary among higher taxonomic groups, median values and overall spread are broadly similar within comparable taxonomic levels. We have updated the Results text and the Figure 2 legend accordingly to reflect this more precise interpretation.

“These patterns in central tendency were broadly consistent across subrealms and taxonomic levels, although distributional shapes varied among higher taxonomic groups (Fig. 2).”

“To evaluate this more formally, we compared distributions across subrealms for groups with the largest sample sizes and found that while distributional shapes varied among higher taxa, median values and overall spread were broadly similar within comparable taxonomic levels (Fig. S2–S4).”

Figure 2 caption: “There were consistent patterns for kingdoms, classes, and orders (B) as shown by similar central tendencies despite variation in distributional shape.”

We refer the Reviewer to the revised manuscript and supplementary material, but show the kindom level in Fig S2.

More broadly, our goal in introducing Species Urbanness Distributions (SUDs) is not to argue that their exact shapes are invariant, but rather to provide a generalizable framework for describing how assemblages are structured along an urbanization gradient. In this respect, SUDs are conceptually analogous to Species Abundance Distributions (SADs), where the precise functional form has long been debated, yet the framework itself has proven extremely valuable for ecology. We therefore emphasize the utility of SUDs as a descriptive and comparative tool for quantifying community-level responses to urbanization, rather than as a claim about strict uniformity in distributional shape across taxa or regions.

**Reviewer #3 (Public review):**

Summary:

This paper reports on an association between body size and the occurrence of species in cities, which is quantified using an 'urban score' that can be visualized as a 'Species Urbanness

Distribution' for particular taxa. The authors use species records from the Global Biodiversity Information Facility (GBIF) and link the occurrence data to nighttime lighting quantified using satellite data (Visible Infrared Imaging Radiometer Suite-VIIRS). They link the urban score to body size data to find 'heterogeneous relationship between body size and urban tolerance across the tree'. The results are then discussed with reference to potential mechanisms that could possibly produce the observed effects (cf. Figure 1).

We thank the reviewer for this clear and accurate summary of the study. We agree that the primary contribution of this work lies in the scale and taxonomic breadth of the analysis, and in introducing a framework (Species Urbanness Distributions) for quantifying species’ relative affinities to urban environments using globally available data. We have revised the manuscript to further clarify the scope of inference and the distinction between descriptive macroecological patterns and mechanistic explanations.

Strengths:

The novelty of this study lies in the huge number of species analyzed and the comparison of results among animal taxa, rather than in a thorough analysis of what traits allow species to persist under urban conditions. Such analyses have been done using a much more thorough approach that employs presence-absence data as well as a suite of traits by other studies, for example, in (Hahs et al. 2023, Neate-Clegg et al. 2023). The dataset that the authors produced would also be very valuable if these raw data were published, both the cleaned species records as well as the body sizes. The paper could strongly add to our understanding of what species occur in cities when the open questions are addressed.

We appreciate highlighting the novelty of the taxonomic breadth and scale of our analysis. We agree that our approach is complementary to more detailed, taxon-specific trait studies based on presence–absence data. In response, we have further emphasized this distinction in the Discussion:

“Our synthesis complements taxon-specific, presence–absence trait studies by identifying broad, cross-taxonomic patterns that can motivate and contextualize more mechanistic analyses17,23.”

We also agree that the cleaned occurrence data and body size information represent a valuable resource, and all data will be made available, with the exception of some body size datasets which we are not able to make available.

Weaknesses:

I value the approach of the authors, but I think the paper needs to be revised.

In my view, the authors could more carefully validate their approach. Currently, any weakness or biases in the approach are quickly explained away rather than carefully explored. This concerns particularly the use of presence-only data, but also the calculation of the urban score.

The vast majority of data in GBIF is presence-only data. This produces a strong bias in the analysis presented in the paper. For some taxa, it is likely that occurrences within the city are overrepresented, and for other taxa, the opposite is true (cf. Sweet et al. 2022). I think the authors should try to address this problem.

We thank the reviewer for raising this important point. We fully agree that GBIF occurrence data are subject to well-known sampling biases, including uneven geographic coverage, observer effort, and taxonomic focus. These limitations are now more explicitly acknowledged in the revised manuscript. At the same time, GBIF currently represents the only global biodiversity database that allows the scope of analysis undertaken here, spanning thousands of species across multiple taxonomic groups and regions. Systematic monitoring datasets that provide presence–absence data are typically restricted to particular taxa (often vertebrates or plants) and are geographically concentrated in the Global North, which would substantially limit the taxonomic and geographic breadth of our analysis.

Importantly, our objective was not to estimate absolute species-specific responses to urbanization, but rather to examine relative patterns of urban affinity across species and families within comparable regional contexts. To address this, we structured our analyses at the subrealm level, which aggregates observations across large spatial extents and reduces sensitivity to fine-scale sampling biases associated with individual cities or urban–rural gradients. In addition, we restricted analyses to species with ≥100 observations per subrealm to focus on well-sampled taxa and reduce the influence of extremely sparse occurrence records. While these steps cannot fully eliminate sampling biases inherent to occurrence data, they substantially mitigate their influence when examining broad comparative patterns.

Recent work has also evaluated the performance of GBIF data in urban biodiversity contexts. For example, Sweet et al. (2022) compared GBIF-derived species richness patterns with independent state-level biodiversity databases across cities and surrounding regions, finding that GBIF provided comparable or broader coverage across taxa and spatial extents. Their analysis showed that species richness was consistently higher in the surrounding region than in the city itself, suggesting that GBIF data capture broad urban–regional biodiversity gradients rather than systematically overrepresenting urban occurrences. Although our analysis differs in design, these results support the use of GBIF as a valuable resource for examining large-scale biodiversity patterns.

More broadly, occurrence databases such as GBIF have become widely used for analyzing species–environment relationships at macroecological scales. While they may be insufficient for estimating precise species-specific environmental tolerances, they are informative for identifying broad patterns across taxa and regions. Our goal here is therefore to identify large-scale comparative patterns in urban affinity and generate hypotheses about trait– urbanization relationships, which can subsequently be tested with more structured monitoring datasets where available.

Another important consideration is that our analyses focus on comparative differences among species within shared taxonomic and geographic contexts, rather than absolute estimates of urban affinity. Sampling biases in occurrence databases are often structured by observer behaviour (e.g., detectability, accessibility, or taxonomic interest), meaning that species recorded by similar observer communities are likely subject to similar sampling biases. Under these conditions, relative differences among species are expected to be preserved even when absolute occurrence frequencies are biased. This logic is consistent with the widely used target-group background approach in presence-only species distribution modelling, where species recorded by similar observer groups (often within the same taxonomic group) are used to control for shared sampling bias. Previous work by Callaghan et al. (2021; https://doi.org/10.1111/gcb.15670) performed additional validation analysis comparing our distribution-based urban affinity metric with estimates derived from occupancy modelling using well-sampled European butterflies (see Fig. S5 from the Callaghan et al. 2021 paper). The strong positive relationship between these approaches suggests that the broad patterns identified here are unlikely to arise solely from sampling artifacts.

Finally, in the revised manuscript we now include additional comparisons among well-sampled taxonomic groups (see responses to other comments throughout our response document for details), which show substantial variation in urban affinity even among taxa with extensive sampling. These results suggest that the patterns reported here are unlikely to arise solely from sampling artifacts, but instead reflect meaningful ecological variation in how species interact with urban environments.

The authors should compare their results to studies focusing on particular taxa where extensive trait-based analyses have already been performed, i.e., plants and birds. In fact, I strongly suggest that the authors should compare their results to previous studies on the relationship between traits, including body size and occurrences along a gradient of urbanisation, to draw conclusions about the validity of the approach used in the current study, which has a number of weaknesses.

We agree that explicitly situating our findings within the existing trait-based urban ecology literature strengthens both interpretation and validation of our approach. We had already referenced several relevant studies (e.g., Hahs et al. 2023 and others) in the Introduction and Discussion, but we recognize that these comparisons were not sufficiently explicit. We have now added text to the Discussion directly comparing our results with previous trait-based studies across taxa:

“Our results are broadly consistent with prior taxon-specific trait-based studies (eg., Hahs et al.[17]), but also highlight that relationships between body size and urbanization vary across taxa and analytical frameworks. For example, global syntheses and regional studies have reported positive, negative, or null size–urbanization relationships depending on clade and spatial scale. A recent global analysis that compiled empirical occurrence data for multiple terrestrial faunal taxa across cities worldwide reported broadly similar body-size responses to urbanization [17]. For four of the five groups that overlap with our analysis—amphibians, bats, bees, and birds—the direction of the body-size relationship with urbanization was consistent between studies. The only exception was carabid beetles, which tended to be smaller-bodied in highly urbanized environments in that analysis, whereas we detected no significant size effect for this family. Studies on birds, for example, have found mixed results, including positive associations to urbanization in some regional assemblages [45], no global relationship in others [46] or an overall negative relationship globally [23], and negative relationships in particular clades such as raptors [40]. Such discrepancies likely arise because different studies quantify urbanization differently, focus on different spatial grains, or analyze different components of species responses (e.g., presence– absence, abundance, or occurrence distributions). Additionally, a study on multiple taxa including butterflies and moths found a positive relationship in butterfly and moth community-weighed mean body size with increases in urbanization level, similar to our findings [31]. Researchers have also found that smaller-bodied dung-associated beetles potentially benefit from urban environments, which is similar to the negative association we found between urbanization and body size in beetles [47]. Our approach complements these studies by estimating occurrence-based urban associations across thousands of taxa simultaneously, allowing comparison of how consistently body size predicts urban affinity across taxonomic groupings rather than within a single lineage. In this sense, variation among published results does not contradict our findings but instead reinforces the conclusion that body size is a context-dependent filter whose direction and strength depend on ecological setting, taxonomic scope, and the urbanization metric used.”

These additions highlight that published relationships between body size and urbanization vary widely across taxa, spatial scales, and analytical approaches. For example, prior studies have reported positive, negative, or null size–urbanization relationships depending on clade, geographic extent, and how urbanization or occurrence is quantified. Even within birds alone, the literature spans positive regional relationships, null global relationships, and negative relationships in particular clades such as raptors. We now explicitly discuss these contrasts and clarify that such discrepancies are expected because different studies measure different components of species’ responses (e.g., presence–absence vs. abundance vs. occurrence distributions), use different spatial grains, or focus on different taxonomic subsets.

We emphasize that our analysis is not intended to replace taxon-specific trait studies, but rather to complement them by providing a macroecological synthesis across thousands of species simultaneously. Importantly, the heterogeneity we observe among families is itself a key biological result, indicating that body size is not a universal predictor of urban affinity but instead a context-dependent filter whose direction and strength vary across ecological and phylogenetic settings. We now state this interpretation more clearly in the revised manuscript.

They should be be more careful in coming up with post-hoc explanations of why the pattern found in this study makes sense or suggests a particular mechanism. This reviewer considers that there is no way in which the current study can disentangle the different possible mechanisms without further analyses and data, so I would suggest pointing out carefully how the mechanisms could be studied.

We agree that our study cannot disentangle the causal mechanisms underlying species’ responses to urbanization. Our intent in discussing potential mechanisms was not to claim definitive explanations, but rather to situate our findings within existing ecological theory and to highlight plausible, non-exclusive pathways that may generate the observed patterns. To make this clearer, we have revised the Discussion to explicitly frame these interpretations as hypotheses rather than conclusions, and to emphasize that testing the underlying mechanisms will require additional data and approaches, such as targeted trait datasets, experimental manipulations, and longitudinal or within-city studies:

“Because our synthesis is correlative and macroecological in nature, the mechanisms discussed above are best viewed as hypotheses that can be evaluated through future work combining experimental, trait-based, and longitudinal data.”.

Additionally, we modified our overall goal to make it clear that this is not inherently a mechanistic study per se:

“Our aim is to identify broad, cross-taxonomic patterns in species’ urban affinity at a global scale, rather than to resolve the specific causal mechanisms driving urban success or failure within individual taxa or cities.”.

More details should be given about the methodology. The readers should be able to understand the methods without having to read a number of other papers.

We have substantially revised and expanded the Methods section to ensure that all analytical steps can be understood directly from the manuscript without requiring consultation of prior publications. In particular, we now (i) provide a clear conceptual roadmap of the workflow at the start of the Methods, (ii) define all key metrics explicitly, including equations for both the urban score and urban affinity, and (iii) clarify the interpretation, assumptions, and limitations of each step. We also added text explaining the rationale for subrealm stratification and the intended interpretation of relative values. Together, these revisions make the methodological framework fully transparent and self-contained (see revised Methods and related responses above and below).

References:

Hahs, A. K., B. Fournier, M. F. Aronson, C. H. Nilon, A. Herrera-Montes, A. B. Salisbury, C. G. Threlfall, C. C. Rega-Brodsky, C. A. Lepczyk, and F. A. La Sorte. 2023. Urbanisation generates multiple trait syndromes for terrestrial animal taxa worldwide. Nature Communications 14:4751.

Neate-Clegg, M. H. C., B. A. Tonelli, C. Youngflesh, J. X. Wu, G. A. Montgomery, Ç. H. Şekercioğlu, and M. W. Tingley. 2023. Traits shaping urban tolerance in birds differ around the world. Current Biology 33:1677-1688.

Sweet, F. S. T., B. Apfelbeck, M. Hanusch, C. Garland Monteagudo, and W. W. Weisser. 2022. Data from public and governmental databases show that a large proportion of the regional animal species pool occur in cities in Germany. Journal of Urban Ecology 8:juac002.

We have incorporated these (and additional new references) into our revised manuscript.

**Recommendations for the authors:**

**Reviewing Editor Comments:**

As you see from the general comments above and the specific recommendations below, the reviewers are impressed by your comprehensive data set and the analytic approach. However, they ask you to clarify your measures of organism size, occurrence data (vs. presence/absence and corresponding sample-bias caveats), urbanness (lighting differences between cities and regions?), urban tolerance (measure should not be relative to other species and particular regions), and region ("subrealm" vs. more commonly used defintions of world regions such as continents). They also encourage you to compare your general results with more detailed local studies to better justify using size as the only, easily available trait.

We thank the Editor for this clear synthesis of the key priorities for revision. We have carefully addressed each point and substantially revised the manuscript to improve clarity, methodological transparency, and interpretability. In particular:

We clarified how body size data were compiled, harmonized, and modeled, including explicit description of how different measurement types (mean, maximum, sex-specific) were retained and statistically accounted for through scaling and hierarchical modeling. We now state these procedures explicitly in the Methods.

We expanded the Methods and Discussion to clarify that our analyses rely on occurrence data rather than presence–absence or abundance data, and we now explicitly discuss the implications and limitations of presence-only datasets, including potential sampling biases and how these may influence inference.

We strengthened justification for using VIIRS night-time lights as a continuous proxy for urbanization, added supporting citations, and clarified that spatial heterogeneity in lighting primarily introduces additional variance rather than systematic bias. We also explicitly describe how urbanization values were calculated and interpreted.

We substantially revised the manuscript to clearly define urban affinity at the outset (including in the Abstract), distinguish it from physiological definitions of tolerance, and provide explicit equations and step-by-step descriptions of how both urban score and urban affinity are calculated and interpreted. We now emphasize that the metric is a relative, region-contextualized measure of occurrence-based urban affinity.

We added full justification, citations, and methodological explanation for the use of biogeographic subrealms, clarified how they differ from continents or climate zones, and explained why this stratification is appropriate for the ecological questions addressed. We also clarified the scope of inference and limitations of this approach.

We expanded the Discussion to explicitly compare our results with prior trait-based urban ecology studies across taxa (including birds and other groups), highlighting where results converge, diverge, and why such variation is expected across spatial scales, taxa, and analytical frameworks.

**Reviewer #1 (Recommendations for authors):**

(1) Abstract

(a) Please define how tolerance is being used here

We now use affinity throughout and it is defined in various places (see responses to other comments here).

(b) The abstract should clarify at what taxonomic scale body size is assessed. It is unclear in the abstract as to whether the reader expects intraspecific measures and interspecific, and at what resolution.

We have revised the abstract by adding one sentence explicitly stating the scale body size was assessed:

“We then assessed whether body size, an integrative ecological trait fundamental to space use, mobility, metabolism, and environmental sensitivity, showed consistent associations with urban affinity among species and across 371 taxonomic families. Analyses were conducted at the interspecific level and focused primarily on variation among taxonomic families (provided with this paper is an accompanying application to view results).”

(2) Results/Discussion

(a) The species urbanness distribution and comparison with the species abundance distribution is an interesting and conceptually useful contribution to urban ecology and underscores how urbanization functions on biodiversity at scale.

We thank the reviewer for this positive assessment and are encouraged that they view the Species Urbanness Distribution (SUD) as a conceptually useful contribution to urban ecology. We see SUDs as a flexible framework that can be extended in several important directions, including comparisons across additional traits, cities of differing size and configuration, and temporal analyses that track how urbanness distributions shift with ongoing urban expansion or restoration. More broadly, we hope that SUDs can provide a framework to think about a macroecological understanding of how urbanization filters biodiversity.

(b) In our Lambert et al. (2023) study that you reference, we suggest that 'exaptation' may be valuable to explore in urban areas. Although body size wasn't the trait we were considering at that time, it may be worth putting your discussion around pre-adaptation in this context.

We agree that exaptation provides a valuable conceptual lens for interpreting species’ responses to urban environments. We have revised the Discussion to explicitly frame species’ urban success in this context:

“Such traits “pre-adapted” to urban conditions allow for some species to not only persist but thrive in urban environments where most species cannot. Framing these patterns through the lens of exaptation may be particularly useful, as traits that evolved under non-urban selective pressures may incidentally confer advantages in urban environments without having arisen in response to urbanization per se (sensu Lambert et al.[4]). We therefore speculate that the skewed shape of SUDs may reflect the uneven distribution of exaptive traits across species pools, rather than widespread adaptive evolution to urban conditions.

Consistent with this interpretation, if exaptive traits that facilitate urban persistence are unevenly distributed across species pools, most species would be expected to exhibit avoidance rather than affinity of urban environments. Indeed, we found that the median urban affinity is most often below one, indicating widespread avoidance among species.”.

(c) Given the family-scale effect, it would be helpful to discuss how often species within a family co-occur in a given geographic region, how much other traits covary with size, etc. Do we have an a priori reason to expect family to be the taxonomic resolution at which body size seems to be most varied?

Our exploratory and preliminary analyses revealed that variation in the body size– urban affinity relationship was strongest at the family level, which prompted us to focus our main analyses at this taxonomic resolution. (But we also present results on order as well). Families represent a biologically meaningful intermediate scale in taxonomy: species within families typically share broad morphological, ecological, and life-history characteristics, yet still exhibit substantial variation in body size and ecological strategies. Indeed, body size is well known to covary with multiple traits—including dispersal ability, metabolism, and space use—making it an integrative trait that captures several ecological dimensions simultaneously within and among families. These correlated traits likely contribute to the heterogeneous responses to urbanization observed among families.

Using the family level also provides a practical balance between biological relevance and statistical robustness. Many families contain sufficient numbers of species to allow independent model estimation while avoiding the strong data imbalance that would arise at higher taxonomic levels. In addition, family is a commonly used unit in macroecological trait analyses (e.g., Roy et al. 2009; Smith et al. 2004), and it often reflects major morphological and ecological similarities among species, as reflected in taxonomic identification frameworks.

Regarding co-occurrence, our analytical framework already accounts for geographic context by estimating urban affinity within subrealms. This ensures that species are compared within the same regional species pools and environmental contexts, rather than across globally disparate assemblages. Consequently, family-level effects emerge from comparisons among species that co-occur within shared biogeographic settings rather than from global taxonomic aggregation.

We have added a short clarification in the manuscript to emphasize that body size functions as an integrative trait that covaries with multiple ecological attributes, and that family-level analyses represent a balance between ecological interpretability and data availability:

“Because body size covaries with multiple ecological traits (e.g., dispersal ability and metabolic rate), we focused on family-level analyses to capture shared ecological strategies while still allowing sufficient variation among species to detect trait– environment relationships [39]”.

(d) The result that body size shows a stronger effect in plants perhaps could suggest that plant records in GBIF are more sensitive to potential collection bias, perhaps due to detectability differences or preferences for where botanists and citizen scientists collect plant data? You mention ornamental plants late, but it may be worth discussing this here, too.

We agree that this is a possible mechanism, which likely conflates detectability and ecological signal. We have expanded this point in the discusssion to better address this:

“These human-driven preferences may also influence detectability and recording effort, as larger and more conspicuous plant species are more likely to be planted, maintained, and documented in urban environments, and thus be available in GBIF for our analyses. However, we suggest that this is not purely a sampling artifact, but such processes likely interact with ecological filtering to shape the realized size structure of urban plant communities.”.

(e) I appreciate the additional taxonomic layering to the discussion. Seeing patterns at the family and order levels is helpful for generating new theory and predictions about how urbanization structures biodiversity at different taxonomic scales.

We agree that examining patterns across multiple taxonomic scales is particularly valuable for generating testable hypotheses about how urbanization structures biodiversity, as different mechanisms may emerge or break down depending on the resolution of analysis. We hope this multi-scale perspective helps stimulate new theory and predictions about the ecological processes shaping urban biodiversity across the tree of life.

(3) Methods

(a) The methodology provides a scalable, consistent, and reasonable measure of both urbanness and species-level urban tolerance. The urban tolerance measure will, of course, not be useful for certain types of research (e.g., animal behavior), but it is appropriate for the resolution of this study.

We agree that the urban affinity metric presented here is intended for broad-scale, comparative analyses and is not designed to capture fine-scale processes such as individual behavior or short-term demographic responses. Our goal was to develop a scalable and consistent measure that enables cross-taxon and cross-region comparisons at a global extent, which we believe is appropriate for addressing the questions posed in this study. We have sought to be explicit about this scope throughout the manuscript (e.g., to better alleviate Reviewer #1 concerns) and emphasize that the framework is complementary to, rather than a replacement for, more mechanistic or organism-focused approaches.

(b) I'm concerned that the authors were not able to constrain their dataset to mean, median, or maximum, not potentially sex variability in sizes. Later in the methods, the authors state that they selected the measure of size that was most common within a family. Does this mean that species within a given family that didn't have that measure of body size were removed from the analysis?

We appreciate this important point and agree that heterogeneity in how body size is measured (e.g., mean, maximum, or sex-specific estimates) is a real and unavoidable challenge in large-scale trait syntheses. Our analytical approach was explicitly designed to minimize the influence of this heterogeneity while retaining as many species as possible, rather than excluding species based on inconsistent trait metadata.

Specifically, species within a family were not removed based on the availability of a particular body size definition. All species with at least one body size estimate were retained. When multiple measures existed for a species, we selected the measurement type that was most commonly available within each family to maximize comparability while preserving sample size. Remaining heterogeneity among measurement types (including units, measurement detail, and whether values reflected means, maxima, or sex-specific estimates) was explicitly accounted for through log-transformation and metadata-aware centering and scaling, with measurement metadata included as random intercepts in the hierarchical models. We have clarified this point in the Methods:

“Importantly, this procedure did not result in the exclusion of species lacking a particular body size definition; rather, all species with at least one available body size estimate were retained, with measurement heterogeneity explicitly accounted for through metadata-aware scaling and hierarchical modeling.”

In addition, our taxonomic modeling strategy was intentionally hierarchical. Species belonging to families that did not meet the minimum threshold for family-level modeling (≥10 species) were not discarded; rather, they were included in higher-level taxonomic analyses (e.g., order- or class-level models), ensuring that available information was retained wherever statistically appropriate. This approach reflects our broader goal of maximizing data inclusion while matching inference to the resolution supported by the data.

**Reviewer #2 (Recommendations for the authors):**

(1) Overlap between VIIRS and GBIF data: While it would have been nice for the GBIF records and VIIRS timescales to match, the degree of mismatch isn't overly large (2010-2021 vs 2015-2021), and any bias or inaccuracies should be minimal. I am mainly making this comment as a potential counterpoint to a possible criticism from other reviewers.

We thank the reviewer for this helpful observation and agree with their assessment. While the temporal coverage of GBIF occurrence records (2010–2021) and VIIRS night-time lights data (2015–2021) does not perfectly overlap, the mismatch is relatively small and unlikely to introduce substantial bias, particularly given our focus on broad, global patterns of urban affinity rather than fine-scale temporal dynamics. We appreciate the reviewer highlighting this point as a potential counterargument to concerns about temporal alignment.

(2) Line 87: "only a select few species seem to possess traits that enable them to thrive in urban...".

This seems like an odd statement, given how many of these species have positive urban tolerance measures.

Agreed that this was oddly worded. We have revised for clarity, focusing on the magnitude of urban affinity:

“Similarly, much like the skewed distributions observed in SADs [24,26], the skewed shape of SUDs indicates that while many species exhibit some degree of urban affinity, a relatively small subset of species attain high levels of urban affinity and dominate urban environments.”

(3) Line 81: "skewed shape of SUDs suggests that traits enabling species to tolerate urban environments are both rare and specific".

Again, based on the shape of some of these curves, I'm not convinced that it is rare, and there is nothing about these curves that suggests it is something "specific". Indeed, urban tolerance could be very multivariate, and the authors' own results suggest this is indeed the case.

We have revised the sentence to retain a focus on traits while avoiding overinterpretation of adaptation from the distributional patterns alone. The revised wording emphasizes the uneven expression of high urban affinity across species without implying rarity or trait specificity:

“The skewed shape of SUDs suggests that traits enabling species to tolerate urban environments are unevenly expressed, given that only a handful of species show extreme urban affinity values, but our results suggest this is geographically widespread across taxa.”.

We also agree with the likelihood that it is multivariate, and return to this in the conclusion in a stronger sense:

“Although body size emerged as a predictor of urban affinity, we found not only substantial heterogeneity across families and orders, but also that body size filtering alone is unlikely to explain the consistently skewed SUD shape. Taken together, these patterns suggest that urban affinity likely emerges from multiple trait combinations rather than a single, universally advantageous trait, and that strong affinity to urban environments is not uniformly expressed across taxa, despite occurring broadly across regions.”.

(4) Line 100: "UHI", avoid abbreviations unless absolutely necessary.

We have removed this abbreviation throughout.

(5) Body size: focusing on one trait seems like a shot in the dark, and so it isn't too surprising that this didn't reveal a strong or consistent pattern. However, I also recognize that collecting consistent trait data across so many taxa is challenging, and size is a low-hanging fruit that correlates with multiple traits. Perhaps discuss more the range of traits you think are most likely to predict urban tolerance.

Body size is indeed the ‘easiest’ to collect, but we acknowledge that there are other traits which could be important, and body size correlates with multiple traits. We revised our discussion to be more comprehensive to discuss some of the additional traits, and be explicit about the shortfalls of body size:

“Ultimately, the heterogeneous and sometimes weak relationships between body size and urban affinity suggests that body size alone cannot explain the emergence of extreme urban exploiters and the skewed shape of SUDs. Focusing on body size as a focal trait necessarily represents a simplification of the multidimensional processes underlying species’ responses to urbanization, driven in part by data availability when conducting a taxonomically-broad synthesis. Instead, urban affinity likely depends on multivariate trait combinations [17,58] that vary among taxa [59] and ecological contexts [60]. Traits that are likely to correlate with urban affinity include dispersal capacity, behavioral flexibility, diet breadth, reproductive strategy, thermoregulatory ability, and, in plants, life history traits such as growth form, clonality, phenology, and seed size. The diversity of trait pathways through which species may persist or thrive in urban environments is consistent with the pronounced taxonomic heterogeneity we observe and helps explain why body size alone does not yield a universal pattern.”

(6) Figure S2: This figure and analysis appear to 'come out of nowhere'. I think this is distracting and tangential, and it should be removed. I have the same thoughts about Figure S3. While I do think a discussion of other traits to measure is well warranted and needed, the inclusion of "preliminary' results that aren't motivated by clear questions, appropriate context, and rigorous analysis should be discouraged.

We have removed Figure S2 and Figure S3 in response to this comment.

I hope the authors find my constructive comments useful in their revision process.

This was a very thorough and thoughtful review. We are greatly appreciative of the opportunity and guidance to improve our work!

**Reviewer #3 (Recommendations for the authors):**

Here is a list of a number of further points that the authors may want to address:

(1) Figure 1 somehow misses the fact that humans simply do not want very large animals in the city. We kill large predators if they come too close to cities, and the same for large herbivores such as wild boar or deer.

We agree that direct human persecution and management of large-bodied species can influence which species occur in urban environments, particularly for large predators and herbivores. Such processes represent important mechanisms shaping urban species assemblages and represent an entire field of socio-ecological dynamics. We have now clarified this point in the Discussion by noting that human–wildlife conflict, management, and persecution could contribute to observed size–urbanization relationships for some taxa, and that disentangling these mechanisms represents an important direction for future research. We added some text to highlight this point:

“Similarly, human–wildlife conflict and active management of large-bodied animals in cities may influence which species persist in urban environments, potentially constraining the upper end of the body size distribution. Taken together, these examples illustrate the importance of considering the socio-ecological context of urban species assemblages [65]”.

(2) Line 270. So you removed all data from the grid-based survey?

We did not remove all data originating from grid-based surveys or gridded products. Rather, we retained GBIF point-occurrence records and applied a standard spatial filtering step, removing only those individual observations with reported coordinate uncertainty greater than 1 km. This was done to ensure reliable alignment between species occurrence points and remotely sensed environmental layers. We have clarified this distinction in the Methods to avoid confusion:

“Due to uncertainty in matching observations with remotely-sensed products, any GBIF observation with a coordinate uncertainty > 1 km was removed. This filtering step removed individual observations with high spatial uncertainty, rather than excluding entire datasets or survey types.”.

(3) Line 278. Human population density?

Yes, we have added ‘human’ here (and elsewhere in this section) to make this clearer to the reader.

(4) Line 284. What is a pixel?

We have modified the text to make this clearer:

“VIIRS Stray Light Corrected Nighttime Day/Night Band Composites product, representing monthly composites, (i.e., this dataset in Google Earth Engine: NOAA/VIIRS/DNB/MONTHLY_V1/VCMSLCFG) with a native resolution of ~500 m^2^. We took the median of all monthly composites for each pixel (i.e., a single grid cell of the night-time lights raster representing a fixed ground area) to calculate a pixel-level urbanization value, measured in average radiance, and used imagery from January 2015 to January 2021 to calculate this median”.

(5) Line 292. It seems to me that lighting is different in different types of cities with the same level of impervious surface, depending on local customs of how many lights are installed, left switched on, etc. I guess that petrol stations and strongly lit industrial areas both produce high levels of light, while for the industrial areas, there could be lawn or other vegetation?

We thank the reviewer for this thoughtful observation and agree that night-time lighting can vary across cities with similar levels of impervious surface due to differences in land use, infrastructure, and cultural lighting practices. We do not interpret VIIRS night-time lights as a direct measure of any single urban feature, but rather as a continuous, integrative proxy for urbanization that captures the combined footprint of human activity, infrastructure intensity, and energy use. VIIRS radiance has been repeatedly shown to correlate strongly with human population density, built infrastructure, and urban extent, while being negatively correlated with vegetation cover (e.g., EVI). It is repeatedly used in remote sensing and urban sustainability literature. This approach is widely supported in the literature, for example:

Panić et al. used night-time lights were to map spatial and temporal patterns of artificial lighting as a proxy for human population distribution and activity, distinguishing areas of urban and rural occupancy. (https://www.ceeol.com/search/article-detail?id=1035395)

Zhou et al. used night-time light observations were to develop a globally consistent time series of annual urban extent, delineating urban clusters and quantifying global urban growth over decades. (https://doi.org/10.1016/j.rse.2018.10.015)

Chakraborty & Stokes used night-time light time series with machine learning to detect and quantify urban change processes—identifying deviations from expected radiance trends to monitor diverse urban transitions. (https://doi.org/10.1016/j.rse.2023.113818)

Zhao et al. reviewed night-time light remote sensing was for its broad capacity to quantify human activities and socioeconomic dynamics—such as urbanization, economic change, and environmental impacts—across scales. (https://doi.org/10.3390/rs11171971)

Zheng et al. used VIIRS nightime lights across 30 global megacities to produce a classification scheme to disentangle urban land changes into five categories, and assess global urbanization processes. (https://doi.org/10.1016/j.isprsjprs.2021.01.002)

Zhao et al. argue that nighttime lights provide a consistent dataset to model and interpret urbanization dynamics and use this to track urban dynamics in Southeast Asia. (https://doi.org/10.1016/j.rse.2020.111980)

While localized mismatches may occur (e.g., brightly lit industrial areas with surrounding vegetation), such heterogeneity is expected to introduce additional variance rather than systematic bias in the measure of urbanization, making our inference conservative. We have clarified this interpretation and added additional supporting references in the Methods:

“Previous work has shown that VIIRS night-time lights is negatively correlated with greenness measured through the Enhanced Vegetation Index (EVI) and positively correlated with human population density [69,71]. Although night-time light intensity can vary among cities with similar impervious surface due to differences in land use, infrastructure, and cultural lighting practices, at broad spatial scales it functions as an integrative proxy of urbanization [75,76,77,78,79,80], with localized heterogeneity contributing primarily to additional variance rather than systematic bias.”

(6) Line 295. How did you reconcile the spatial uncertainty of >1km with an urbanization pixel of 150m2? For how many species did you have a higher uncertainty than pixel size? In my experience, your ca. 39m accuracy is a strong assumption for GBIF data.

We would like to clarify that we do not assume species occurrence accuracy at the scale of the geohash blocks (i.e., tens of meters), and we do not interpret GBIF records as having ca. 39 m positional accuracy. The use of geohash7 (~150 m blocks) reflects a computational indexing choice, not an assumption about biological or observational precision. All GBIF observations with reported coordinate uncertainty greater than 1 km were removed prior to analysis, ensuring that retained occurrences were compatible with the effective spatial resolution of the remotely sensed urbanization data. Importantly, the effective spatial resolution of our urbanization metric remains that of the VIIRS night-time lights product (~500 m). Geohash encoding at a finer resolution was used solely to efficiently associate point occurrences with the appropriate VIIRS pixel while avoiding redundant extraction or averaging across adjacent pixels. This approach does not increase the effective spatial precision of the analysis, nor does it imply sub-pixel inference. We have clarified this in the Methods:

“The VIIRS night-time lights data, with a native resolution of ~500 m^2^, was then matched to these blocks by assigning each geohash7 block the average VIIRS radiance value that intersects it. We do not assume positional accuracy at the scale of the geohash blocks, but geohash encoding was used solely for computational indexing, while the effective spatial resolution of the urbanization metric is that of the VIIRS data (~500 m). This approach allows us to avoid unnecessary redundancy in the data while maintaining the original VIIRS resolution”.

(7) Line 296. Why this high resolution in the species data when your light data is 500m2?

The apparent mismatch in resolution reflects a distinction between data handling resolution and analytical resolution. Species occurrence records were retained at their native point-level precision to avoid premature spatial aggregation and to ensure that each observation could be accurately matched to the appropriate VIIRS night-time lights pixel. The finer-resolution geohash encoding does not imply that species data were analyzed at that scale, nor does it increase the effective spatial resolution of the analysis. We note, however, that the reported spatial uncertainty of some GBIF records may approach or exceed the resolution of the VIIRS data. Retaining such records represents a deliberate trade-off between spatial precision and data coverage, and is necessary to maximize taxonomic and geographic representation in a global analysis of this scope. Importantly, any residual spatial uncertainty is expected to introduce additional noise rather than systematic bias, making our estimates of species–urban affinity relationships conservative.

(8) If you could show how your results match the results of Hahs et al and others with respect to occurrence and traits, this would strengthen your approach.

We agree that explicitly comparing our findings with prior trait-based studies strengthens the interpretability of our approach. We have now added text to the Discussion that directly compares our results with published analyses, including Hahs et al. (2023) and other taxon-specific studies. In particular, we highlight where our occurrencebased estimates recover similar body size–urbanization relationships (four of five taxa in Hahs et al.) and where they differ (e.g., carabids), and we discuss how such differences likely arise from variation in spatial grain, response variables, and definitions of urbanization. These additions clarify how our framework aligns with, complements, and extends existing trait-based work rather than replacing it.

(9) I wonder whether you could run your analysis with simplified data. In the end, you do not talk much about how high the urban score is, so you may also aggregate values to "highly lighted", "lighted", "some light" and "dark" and re-do the analysis, after checking how these scores correlate with e.g. impervious surface in a slightly larger area than what you used (maybe 50x50m).

Our analytical framework—and the concept of Species Urbanness Distributions (SUDs) in particular—relies on retaining the continuous nature of the underlying urbanization metric. Discretizing night-time light values would necessarily introduce arbitrary thresholds, reduce information content, and obscure subtle but ecologically meaningful variation in species’ relative affinities to urban environments. Because we focus on relative affinity patterns rather than absolute urbanization classes, maintaining a continuous metric is central to both our methodological approach and conceptual contribution. That said, we agree that exploring how continuous urban affinity scores relate to categorical urban classes or alternative urbanization proxies (e.g., impervious surface at different spatial grains) represents a valuable direction for future work. Such analyses could be particularly informative for translating continuous affinity metrics into applied conservation or urban planning contexts.
